# Proinflammatory Matrix Metalloproteinase-1 Associates With Mitral Valve Leaflet Disruption Following Percutaneous Mitral Valvuloplasty

**DOI:** 10.3389/fcvm.2021.804111

**Published:** 2022-01-20

**Authors:** Livia S. A. Passos, Dakota Becker-Greene, Renato Braulio, Thanh-Dat Le, Cláudio L. Gelape, Luís Felipe R. de Almeida, Divino Pedro A. Rocha, Carlos Augusto P. Gomes, William A. M. Esteves, Luiz G. Passaglia, Jacob P. Dal-Bianco, Robert A. Levine, Masanori Aikawa, Judy Hung, Walderez O. Dutra, Maria Carmo P. Nunes, Elena Aikawa

**Affiliations:** ^1^Center for Excellence in Vascular Biology, Division of Cardiovascular Medicine, Department of Medicine, Brigham and Women's Hospital, Harvard Medical School, Boston, MA, United States; ^2^School of Medicine, Hospital das Clínicas, Federal University of Minas Gerais, Belo Horizonte, Brazil; ^3^Cardiac Ultrasound Laboratory, Massachusetts General Hospital, Harvard Medical School, Boston, MA, United States; ^4^Center for Interdisciplinary Cardiovascular Sciences, Division of Cardiovascular Medicine, Department of Medicine, Brigham and Women's Hospital, Harvard Medical School, Boston, MA, United States; ^5^Department of Human Pathology, Sechenov First Moscow State Medical University, Moscow, Russia; ^6^Departamento de Morfologia, Instituto de Ciências Biológicas, Universidade Federal de Minas Gerais, Belo Horizonte, Brazil; ^7^Instituto Nacional de Ciência e Tecnologia em Doenças Tropicais, Belo Horizonte, Brazil

**Keywords:** percutaneous mitral valvuloplasty, MMP-1, collagen, inflammation, rheumatic heart disease

## Abstract

Mitral regurgitation (MR) is a major complication of the percutaneous mitral valvuloplasty (PMV). Despite high technical expertise and cumulative experience with the procedure, the incidence rate of severe MR has not decreased. Although some of MR can be anticipated by echocardiographic analysis; leaflet tearing, which leads to the most dreaded type of MR, remains unpredictable. Irregular valvular collagen remodeling is likely to compromise tissue architecture and increase the tearing risk during PMV balloon inflation. In this study, we evaluated histological and molecular characteristics of excised mitral valves from patients with rheumatic mitral stenosis (MS) who underwent emergency surgery after PMV due to severe MR caused by leaflet tear. Those findings were compared with patients who underwent elective mitral valve replacement surgery owing to severe MS, in whom PMV was not indicated. *In vitro* assay using peripheral blood mononuclear cells was performed to better understand the impact of the cellular and molecular alterations identified in leaflet tear mitral valve specimens. Our analysis showed that focal infiltration of inflammatory cells contributes to accumulation of MMP-1 and IFN-γ in valve leaflets. Moreover, we showed that IFN-γ increase the expression of MMP-1 in CD14^+^ cells (monocytes) *in vitro*. Thus, inflammatory cells contribute to unevenly remodel collagen resulting in variable thickening causing abnormalities in leaflet architecture making them more susceptible to laceration.

## Introduction

Hemodynamically significant mitral regurgitation (MR) can be a major complication when treating rheumatic mitral stenosis (MS) with percutaneous mitral valvuloplasty (PMV) (1). Despite high technical expertise and cumulative experience with PMV, the incidence rate of severe MR has not decreased (2, 3). As the rheumatic MS disease process can cause distinct structural valvular derangements, the pattern of extracellular leaflet matrix changes and thickening may predict the risk of MR following PMV (4, 5).

Different mechanisms are involved in the development of MR after PMV. Although some of them can be anticipated by echocardiographic analyses, leaflet tearing, which leads to the most dreaded type of MR, remains unpredictable. Over the last several decades, the incidence of severe MR owing to leaflet rupture remains unchanged and up to 2% of patients may require urgent mitral valve (MV) replacement (1, 6). As patients with rheumatic MS are usually young, mechanical prostheses are favored over tissue prostheses as they only have limited durability requiring repeated surgeries. A mechanical prosthesis, however, has the risks of coumadin related bleeding, stroke, endocarditis, and prosthesis malfunction as well as pregnancy and delivery complications. Therefore, it is essential to determine the underlying mechanisms that are potentially responsible for valve/leaflet integrity disruption after PMV. In this context, comprehensive histological evaluation of the excised MVs from patients who developed post-PMV severe MR due to leaflet tearing may provide novel insights into such mechanisms.

The MV leaflets are covered by endothelium and composed of three layers of specialized extracellular matrix (ECM) containing heterogeneous valvular interstitial cells (7, 8). Rheumatic heart disease leads to a substantial changes in MV leaflet architecture due to alteration of its matrix and cellular components (5). Inflammation and subsequent ECM remodeling are likely compromising the physiologic range of leaflets tissue properties increasing the tearing risk during PMV balloon inflation. Moreover, it is likely that inflammation and remodeling “pockets” are localized/heterogeneous within leaflets and with differing severity levels. Fibrotic valvular remodeling is essential for wound healing in response to tissue injury and is characterized by the accumulation of collagen and other ECM components (9). Matrix metalloproteinases (MMPs) are matrix-degrading enzymes that play a pivotal role in cardiovascular pathophysiology by degrading matrix or non-matrix substrates (10, 11). MMPs, immune cells and fibroblasts regulate their functions jointly, influencing disease outcome (11, 12). Proteolytic imbalance can provide significant insights into the progression of aberrant valvular remodeling and post-procedural leaflet tear susceptibility.

In this study, we evaluated histological and molecular characteristics of excised MVs from patients with rheumatic MS who underwent emergency surgery after PMV due to severe MR caused by leaflet tearing. The histological findings were compared with patients who underwent elective MV replacement surgery owing to severe MS, in whom PMV was not indicated.

## Materials and Methods

### Study Population and Echocardiographic Assessment

**Figure 1:**
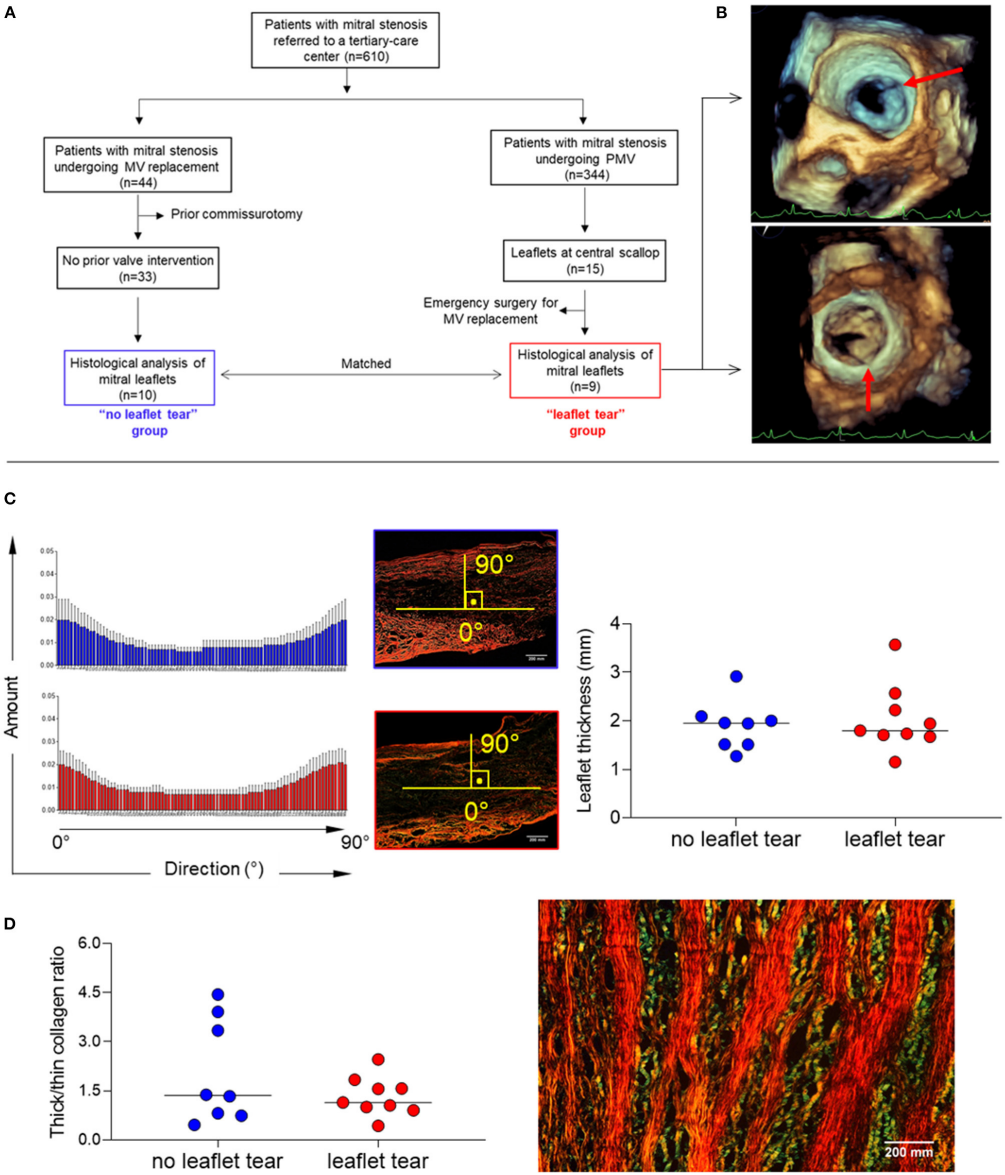
**(A)** Diagram of total study population selection and groups definition (“leaflet tear” group *n* = 9 and “no leaflet tear” group *n* = 10). **(B)** Representative 3D TEE showing leaflet tear (arrows) during PMV. **(C)** Analysis of patterns of collagen fiber orientation (left panel) and measure of MV leaflet thickness (right panel), using picrosirius red staining in “no leaflet tear” (*n* = 8) and “leaflet tear” (*n* = 9) groups. The histograms report the percentage of collagen fibers aligned within a specific orientation from the longitudinal direction. An orientation of the collagen fibers parallel to the axis bundle corresponds to 0°, and an orientation in a transverse plane correspond to 90°. For each orientation, the mean and standard deviation are reported among all the axial section of each patient. **(D)** Analysis of collagen composition through thick/thin collagen ratio (left panel) in “no leaflet tear” (*n* = 8) and “leaflet tear” (*n* = 9) groups, using picrosirius red staining and representative image of thick and thin collagen layers in stenotic MVs (right panel). The color changes from green to yellow to orange to red as thickness increases. Scale bar = 200 mm.

Of 344 patients who underwent PMV for significant rheumatic MS between 2011 and 2019, a total of 9 patients (2.9%) developed severe MR due to leaflet tearing (“leaflet tear” group). These patients were compared with 10 patients who were unsuitable for PMV and underwent valve replacement to treat MS; they were matched by age, sex, valve morphology and stenosis severity (“no leaflet tear” group). The flow chart of the study population selection is shown in Figure 1A.

A standard transthoracic two-dimensional (2D) echocardiography was performed using commercially available equipment (EPIQ with an X7-2t TEE probe, Philips Medical Systems, Andover, MA), and all measurements were made according to recommendations of the American Society of Echocardiography (13). Three-dimensional (3D) transesophageal echocardiography (TEE) was also performed in all patients before and immediately after PMV for a comprehensive evaluation of the MV (Figure 1B). MV leaflet tearing with severe MR was defined as a tear either in the anterior or posterior leaflets at central scallop location (A2/P2) or a combination of lesions including both leaflet laceration without commissural involvement. PMV was performed using Inoue technique guided by a transthoracic echocardiography to assess valve orifice area by planimetry and the degree of MR after each balloon dilatation (14). The technique consisted of advancing a catheter over the wire across the interatrial septum and advancing one large balloon (Inoue balloon) across the mitral orifice and inflating it within the orifice with gradual increase of balloon size on sequential inflations. Before placing the Inoue balloon catheter, the balloon size was estimated based on patient height. After each dilatation, the balloon size was increased with an additional 1 ml increment in the balloon volume was made until an adequate valve area has been achieved or an increase in MR was seen.

This study was approved by the Brazilian National Institutional Review Board (CONEP), number 32715214.9.0000.5149.

### Histopathological Assessment

Middle portions of anterior fragments were collected from patients who underwent MV replacement due to rheumatic MS from both “no leaflet tear” and “leaflet tear” groups and then embedded into Optimum Cutting Temperature compound (OCT, Tissue-Tek®, 4583). The cryoblocks were sectioned into 7 μm slices using a cryostat (Leica CM3050S) followed by histological, immunohistochemical and immunofluorescence staining.

### Hematoxylin and Eosin Staining

MV sections were fixed for 20 mins in 10% formalin, then stained with successive baths of Harris hematoxylin for 1 min and alcoholic eosin for 1 min to assess overall morphology.

### Masson's Trichrome Staining

MV sections were fixed 30 mins in Bouin solution, then stained with sequential solutions of Weigert iron hematoxylin for 5 mins, Biebrich Scarlet fuchsin for 10 mins, phosphomolybdic acid for 30 s, aniline blue for 3 mins and acetic acid for 30 s. MV leaflet thickness was measured for each leaflet ten times across areas free of chordal attachments using ImageJ software (NIH); all values are averaged and presented in mm.

### Picrosirius Red Visualized Under Polarized Light Microscopy

MV sections were fixed 10 min in 10% formalin, and then dipped in a solution of picric acid 0.1% sirius red for 3 h, followed by HCl 0.01N solution for 1 min. For collagen orientation analysis, cryosections were stained with picrosirius red viewed with circularly polarized light to assess collagen architecture, orientation and organization (15). We assessed the proportion of different colored fibers using a polarized light microscope (Nikon), and measured collagen composition in the whole section of MV leaflet by ImageJ software (NIH). To measure ECM collagen fiber orientation, 3 pictures of 200× magnification were taken and analyzed for each valve. Colored images were transformed into gray scale, analyzed with ImageJ using Directionality plugin, which exploits the Local Gradients orientation method.

### Immunohistochemistry

MV inflammatory infiltrates were identified by immunohistochemistry using markers for mononuclear cell subtypes. Cryosections were fixed in −20°C acetone (Fisher Scientific, MA, USA), blocked with 0.3% hydrogen peroxidase (Fisher Scientific, MA, USA) and incubated with Protein Block Serum-Free (DAKO, CA, USA). Primary antibodies anti-CD68 (human, 1:500, DAKO, CA, USA), anti-CD4 (human, 1:30, Fisher Scientific, USA) and anti-CD8 (human, 1:80, Fisher Scientific, USA) were diluted in 5% inactivated normal horse serum (Vector Laboratories, CA, USA). Sections were incubated with primary antibodies for 90 mins at 4°C overnight followed by 45 mins incubation with biotinylated goat anti-mouse and anti-rabbit secondary antibodies (Dako, LSAB Kit). The streptavidin peroxidase method (Dako, LSAB Kit) was performed for each staining, and the reaction was visualized with a 3-amino-9-ethylcarbazol substrate (AEC Substrate Chromogen, Dako). Sections were counterstained with Gill's No. 3 Hematoxylin (Sigma-Aldrich, MO, USA). For quantification, AEC-positive cells (red reaction product) and total nuclei (blue) per high-power field (400× magnification) were counted and expressed as AEC-positive cells/nuclei for each marker. Ten fields were quantified for each section.

### Real-Time Quantitative Polymerase Chain Reaction

Human MVs embedded in OCT compound were used to obtain samples for real-time quantitative polymerase chain reaction. For each specimen, 5 sections of 10 μm were transferred to pre-chilled 1.5 ml microcentrifuge tubes, suspended in 1 ml TRIzol™ reagent (Ambion, 15596018) and stored for ~2 weeks at −80°C. Total RNA was isolated per manufacturers' instructions. Briefly, 0.2 ml chloroform was added to each sample and homogenized. After a 2-min incubation at room temperature (RT), samples were centrifuged at 12,000 × g, 4°C for 15 mins using an Eppendorf 5430R microcentrifuge. The aqueous (upper) phase of each sample was then transferred to a new 1.5 ml tube. Next, 0.5 ml of isopropanol and 1.5 μl of GlycoBlue™ coprecipitant (Invitrogen™, LSAM9515) were added to each sample, followed by sample homogenization, incubation at RT for 10 mins, and centrifugation at 12,000 × g, 4°C for 10 mins. After discarding the supernatant, RNA pellets were washed using 75% ethanol, mixed using a vortex, and centrifuged at 7,500 × g, 4°C for 5 mins. The supernatant was discarded, RNA pellets were air dried for 10 mins, and resuspended in 10 μl RNase-free water. All purified RNA samples were stored at −80°C.

RNA concentration was quantified using the NanoDrop™ 2000 spectrophotometer (Thermo Fisher™, ND-2000). Next, cDNA was prepared using 200 ng of RNA per sample with 5 × EasyQuick RT MasterMix (CoWin Biosciences, CW2019M) per manufacturer's protocol. In brief, 200 ng RNA was transferred to a clean 8-strip 200 ul tube and volume was brought to 16 μl total using RNase-free water. Then, 4 μl 5X EasyQuick RT MasterMix was added to each sample, mixed thoroughly, and centrifuged briefly to ensure collection of all solution at the bottom of the tube. Reverse-transcription performed using a PCR Thermal Cycler T100™ (Bio-Rad Laboratories, 1861096) set at 37°C for 15 mins, followed by incubation at 85°C for 5 mins. Prepared cDNA diluted 1:4 using RNase-free water and stored at −20°C. PerfeCTa® SYBR® Green FastMix®, ROX (Quantabio, 95073-05K) was used for quantitative real-time qPCR analysis with the 7900HT Fast Real-Time PCR System (Applied Biosystems™, 4329001) following the manufacturer's instructions. Gene-specific primers were used to detect human MMP-1, MMP-8, MMP-10, MMP-13, MMP-14, IL-17A, TIMP-1, IFN-γ. Samples were normalized to endogenous human HPRT. Fold changes were calculated by ΔΔCt method. Data sets did not assume Gaussian distribution; the difference was determined by Mann-Whitney with *p* < 0.05 considered significant.

### *In vitro* Peripheral Blood Mononuclear Cells Stimulation With Recombinant IFN-*Γ* (RIFN-*Γ*)

PBMCs (Lonza, USA) from healthy donors were plated at 1 × 10^6^ cells/well in 96 well cell culture round bottom plates (Corning, USA) with RPMI media (Thermo Fisher Scientific, USA) supplemented with antibiotic (penicillin 200 U/ml and streptomycin 0.1 mg/ml; Sigma, USA) and 1 mM L-glutamine (Sigma, USA) and 5% inactivated human serum (Sigma, USA) in the presence 5 ng/ml of human rIFN-γ (I17001, Sigma, USA) or media only (non-stimulated) for 12 h (37°C, 5% CO_2_). Then, cell cultures were centrifuged at 600 g for 8 mins, at 4°C. Brilliant buffer solution (100 ul) (BD, USA) containing monoclonal antibodies specific for human leukocyte cell-surface markers were added to the cells, including anti-CD8 (APCCy7, Biolegend, USA), anti-CD4 (PerCP/Cyanine5.5, Biolegend, USA) and anti-CD14 (PECy7, Biolegend, USA) to identify T-cells subpopulations and monocytes, respectively. Expression of MMP-1 in those cells were accessed by using anti-MMP-1 (PE, R&D Systems, USA). PE isotype control was used to confirm the lack of non-specific staining for MMP-1. Zoombie Aqua fixable viability kit was used to access cell viability (BV510, Biolegend, USA). Samples were washed two times in flow cytometry staining buffer (FACSbuffer, eBioscience, USA) and read in a BD LSR II Flow Cytometer. Samples containing 50,000 cells were collected. FACS data were analyzed using FlowJo (FlowJo, USA). Paired T test was used to compare unstimulated vs. stimulated cultures. *P* ≤ 0.05 were considered statistically significant. T-distributed stochastic neighbor embedding (t-SNE) unsupervised analysis was performed to segregate cell populations using Cytofkit package (16).

### Statistical Analysis

D'Agostino, Shapiro-Wilk and Kolmogorov-Smirnov tests were used to verify the dataset normality for all performed assays. In the histological and *in vitro* analyses, the group values assumed Gaussian distribution. Unpaired *t* test was used to ascertain differences between “no leaflet tear” and “leaflet tear” groups. Paired *t* test was used to ascertain differences between “non-stimulated” and “rIFN-γ stimulated” cell cultures. As qPCR datasets did not assume Gaussian distribution, Mann-Whitney test was used to determine differences between “no leaflet tear” and “leaflet tear” groups. Correlation analyses were done using the Pearson correlation coefficient. Differences that returned *p* ≤ 0.05 were considered statistically significant. All analyzes were performed using the Prism 8 software (GraphPad Software).

## Results

### Baseline Clinical Characteristics

Clinical characteristics of the study population according to the presence of leaflet tear are presented in Table 1. Overall demographic and clinical features were similar between the patients with and without leaflet tear. All patients had severe rheumatic MS with no differences related to the MV morphology by echocardiography.

**Table 1:** Baseline clinical characteristics stratified according to the presence of leaflet tear.

	**No leaflet tear** **(***n*** = 8)**	**Leaflet tear** **(***n*** = 10)**	***p*** **value**
Age (years)	44.3 ± 14.1	50.8 ± 9.1	0.249
Female gender (%)	8 (100)	9 (90)	0.357
Body surface area (m^2^)	1.68 ± 0.2	1.83 ± 0.3	0.332
Atrial fibrillation	1 (13)	2 (20)	0.671
NYHA Functional class III/IV	4 (50)	6 (60)	0.395
LV ejection fraction (%)	60.2 ± 1.3	63.7 ± 8.9	0.253
LA dimension (mm)	52.3 ± 2.5	51.9 ± 7.6	0.401
LA volume (ml/m^2^)	57.7 ± 14.1	53.9 ± 17.1	0.676
Peak trans mitral gradient (mmHg)	19.4 ± 8.1	18.0 ± 5.6	0.682
Mean trans mitral gradient (mmHg)	10.4 ± 5.4	10.2 ± 3.6	0.934
Mitral valve area (cm^2^)	1.2 ± 0.5	1.1 ± 0.2	0.504
SPAP (mmHg)	55.8 ± 23.6	42.9 ± 9.6	0.135
Right ventricular FAC (%)	43.8 ± 9.9	47.8 ± 10.8	0.553
Leaflet calcification	3 (38)	1 (10)	0.113
Echocardiographic score	8.6 ± 1.1	7.6 ± 1.5	0.216
C_n_ (ml/mmHg)	4.5 ± 1.9	4.9 ± 1.8	0.696

*C_n_ = net atrioventricular compliance; LA, center atrium; LV, center ventricle; NYHA, New York Heart Association; RVFAC, right ventricular fractional area change; SPAP, systolic pulmonary artery pressure*.

### Stenotic Mitral Valves Leaflets Display Alternating Thick and Thin Collagen Layer Structure

Alignment of collagen fibers and leaflet thickness were similar between the groups (Figure 1C). The quantification of thick and thin collagen fibers was not different between the groups (Figure 1C). A clear architectural pattern of valve tissue collagen organization was observed in all specimens, in which thick collagen (red/orange) and thin collagen (green) fibers alternated (Figure 1D). Layered soft tissue interspersing with fibrous tissue was present which could predispose to leaflet tear in the softest areas.

### Inflammatory Infiltrates Are Localized in Leaflet Low Density Collagen Areas

**Figure 2:**
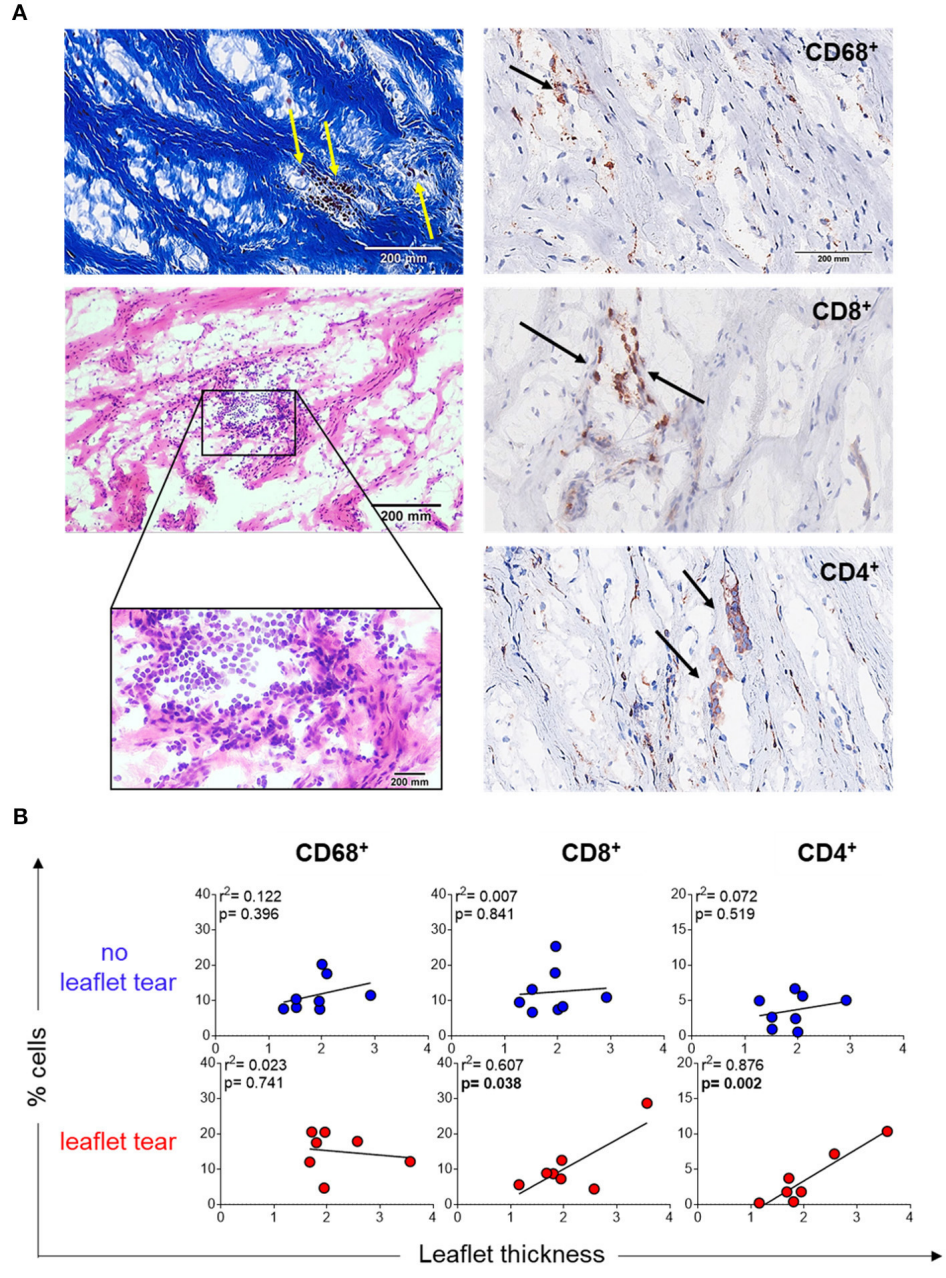
Inflammatory infiltrate in stenotic mitral valves. **(A)** Representative Masson's trichrome (upper panel) and hematoxylin and eosin (bottom panels) staining showing clusters of mononuclear inflammatory cells localized in low-density collagen areas (left). Representative images for immunohistochemistry staining for CD68, CD8, and CD4 stenotic mitral valves (right). Graph shows the frequency of each cell subset from patients in no leaflet tear group (blue, n=8) and in leaflet tear group (red, *n* = 8). Scale bar = 200 mm. **(B)** Correlation analysis between the frequency of CD68, CD8, and CD4 and leaflet thickness in rheumatic mitral valves in no leaflet tear group (blue, *n* = 8) and in leaflet tear group (red, *n* = 7).

Another explanation of thin collagen fiber accumulation could be due to the focal infiltration of inflammatory cells, which may unevenly remodel collagen resulting in variable thickening. Stenotic MV leaflets showed mononuclear cell infiltrates in focal areas along collagen fibers as shown by Masson's trichrome (left top panel) and hematoxylin and eosin (left low panel) staining (Figure 2A). Notably, these cells were predominantly colocalized within the areas of low collagen density (weak Masson's staining) (Figure 2A). Immunohistochemistry quantification of macrophages (CD68^+^), T helper cells (CD4^+^) and T cytotoxic cells (CD8^+^) confirmed the localization of those cells in low density collagen areas (Figure 2B). MVs from the “no leaflet tear” and “leaflet tear” groups did not show any difference in the frequency of these inflammatory cells (Supplementary Figure 1). A moderate positive correlation was observed between the CD4^+^ and CD8^+^ T-cell frequency and valvular thickness in the “leaflet tear” group (Figure 2B). CD4^+^ T-cells showed stronger correlation with leaflet thickness (*p* = 0.002, *r*^2^ = 0.876) as compared to CD8^+^ T-cells (*p* = 0.038, *r* ^2^ = 0.607). No association of leaflet thickness with CD68^+^ (macrophages) cells was found (Figure 2B).

### MMP-1 Is Focally Highly Expressed in MV Leaflets From Patients With Leaflet Tear

**Figure 3:**
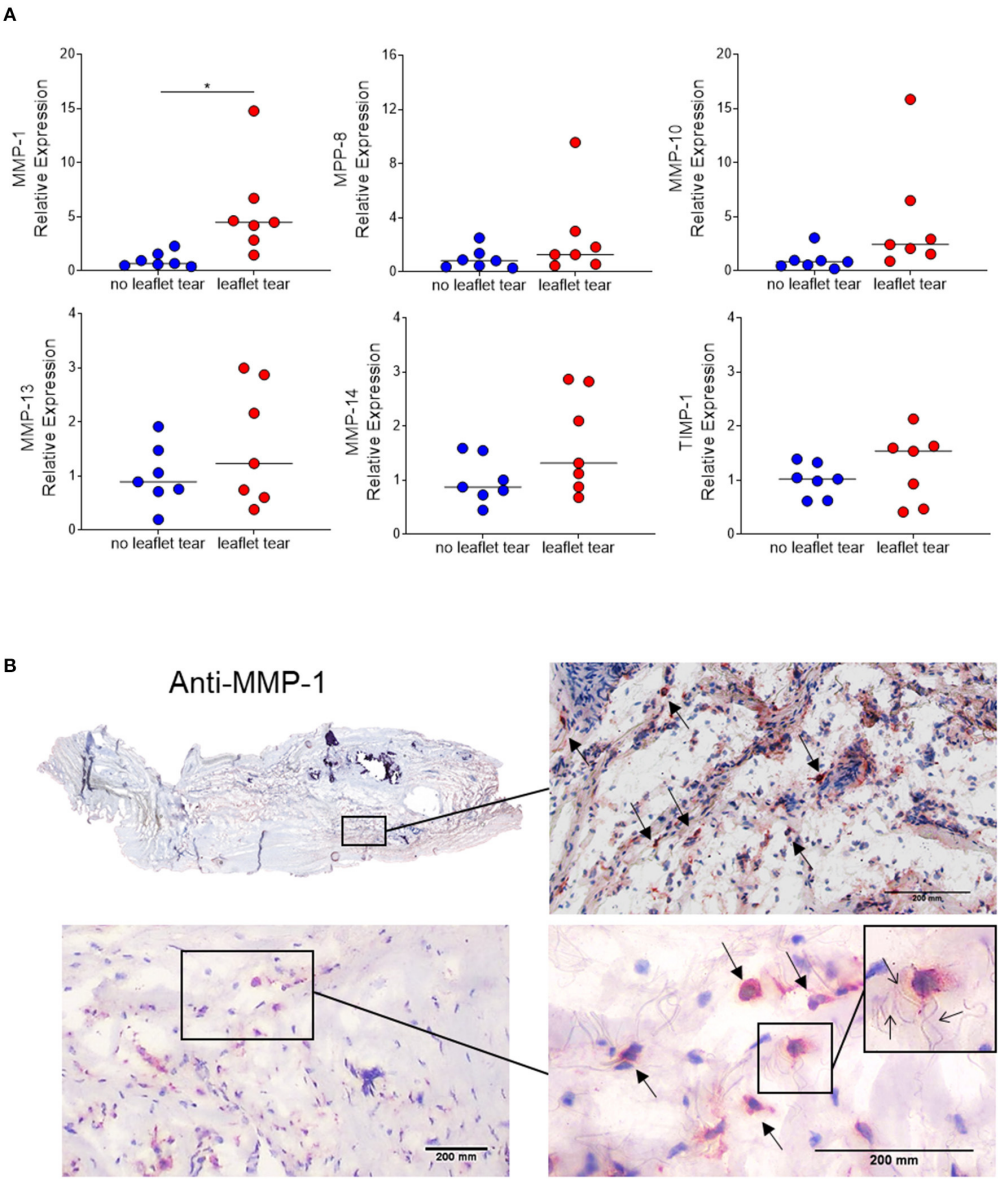
**(A)** mRNA relative expression of matrix metalloproteinases (MMP) 1, 8, 10, 13, 14 and tissue inhibitor metallopeptidase 1 (TIMP-1) in “no leaflet tear” group (blue, *n* = 7) and in “leaflet tear” group (red, *n* = 7) by real-time quantitative polymerase chain reaction. Graph shows the individual values and median of mRNA relative expression for each group. **(B)** Entire leaflet section (upper left) representative images for immunohistochemistry staining for MMP-1. Boxed higher-magnification images show inflamed areas with cells expressing MMP-1 (arrows). Scale bar = 200 mm.

Since inflammatory infiltrates were mainly observed in low density collagen areas, we sought to evaluate the expression of matrix metalloproteinases (MMPs), the major contributors to collagen degradation. MMP-1 mRNA expression levels in the “leaflet tear” group were significantly higher (5.5-fold; *p* = 0.017) than in the “no leaflet tear” group. Albeit statistically not significant, MMP-8, MMP-10, MMP-13 and MMP-14 were 2.5-fold (*p* = 0.220), 4.6-fold (*p* = 0.100), 1.6-fold (*p* = 0.244) and 1.6-fold higher (*p* = 0.097), respectively, in the “leaflet tear” group (Figure 3A). We also evaluated the expression of tissue inhibitor metallopeptidase 1 (TIMP-1) and found a non-significant 1.25-fold increase in the “leaflet tear” vs. the “no leaflet tear” group (*p* = 0.386) (Figure 3A). We further evaluated the spatial distribution of MMP-1 by immunohistochemistry. MMP-1 was focally expressed in the leaflet areas rich in inflammatory infiltrates, suggesting that these cells are a likely source of MMP-1 (Figure 3B). We also observed disruption and disorganization of collagen fibers around the MMP-1 positive cells (Figure 3B). Taken together, these findings suggest that increased MMPs, particularly MMP-1 levels, could contribute to leaflet tearing by potentially weakening the leaflet tissue.

### Elevated MMP-1 Correlates With Increased IFN-*Γ* Expression in the Leaflet Tear Group

**Figure 4:**
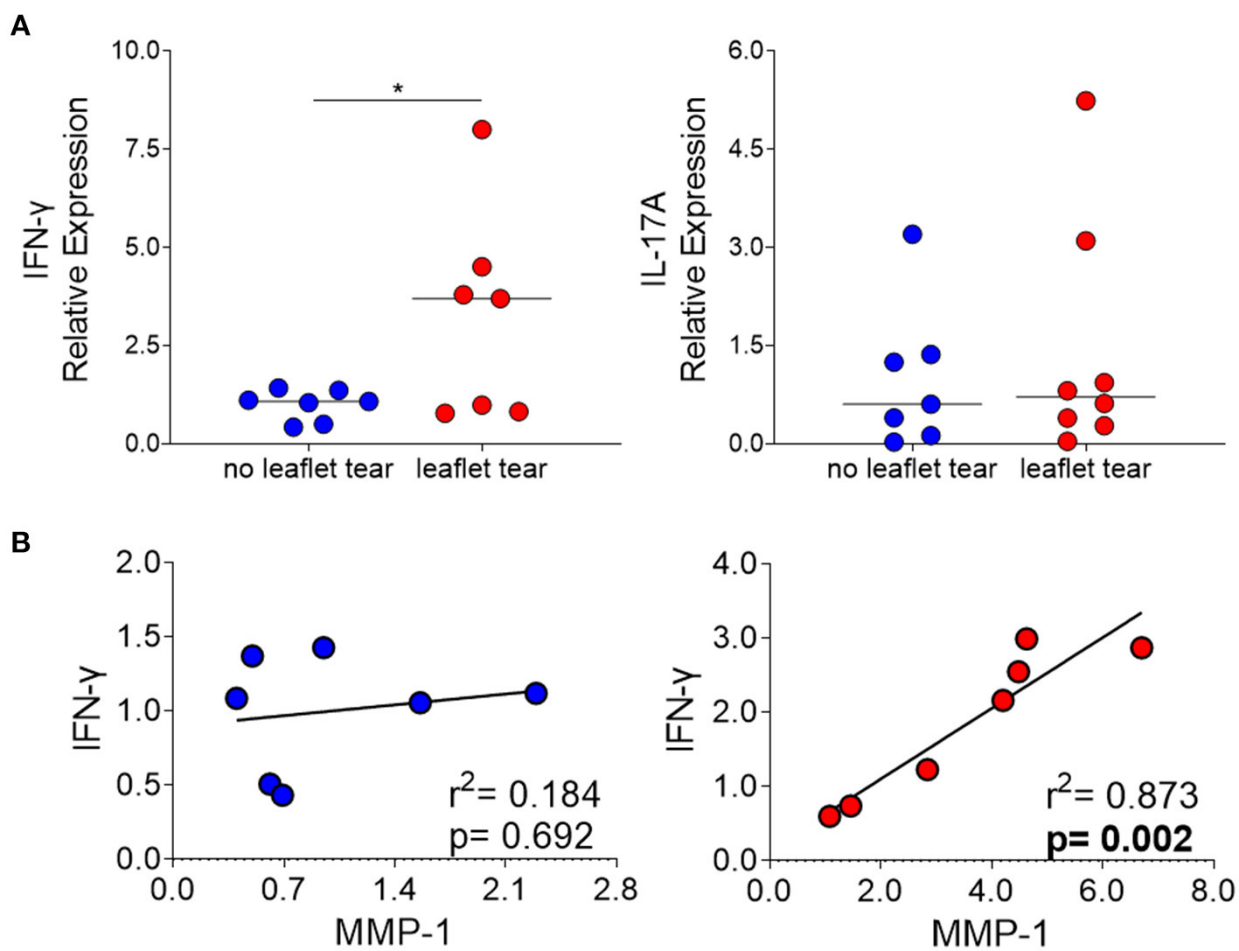
**(A)** mRNA relative expression of cytokines IFN-γ and IL-17 in “no leaflet tear” group (blue, *n* = 7) and in “leaflet tear” group (red, *n* = 7) by real-time quantitative polymerase chain reaction. Graph shows the individual values and median of mRNA relative expression for each group. **(B)** Correlation analysis between mRNA relative expression of IFN-γ and MMP-1 in “no leaflet tear” group (blue, *n* = 8) and in “leaflet tear” group (red, *n* = 7). *means statistically significant.

We also evaluated the mRNA expression of interferon gamma (IFN-γ) and interleukin 17A (IL-17A) in “no leaflet tear” and “leaflet tear” groups since these cytokines play roles in T cell-mediated proinflammatory responses and ECM remodeling. We found a significant 3.2-fold increase in the mRNA expression levels of IFN-γ in the “leaflet tear” group as compared to “no leaflet tear” (*p* = 0.046), while IL-17A showed only a modest 1.43-fold increase (Figure 4A). A strong positive correlation between IFN-γ and MMP-1 in the “leaflet tear” group indicated an association between these two proinflammatory factors (*p* = 0.002, *r*^2^ = 0.873) (Figure 4B). These results suggest that IFN-γ may contribute to increased MMP-1 expression.

### *In vitro* PBMC Stimulation With RIFN-*Γ* Increases MMP-1 Expression in CD14^+^ Cells

**Figure 5:**
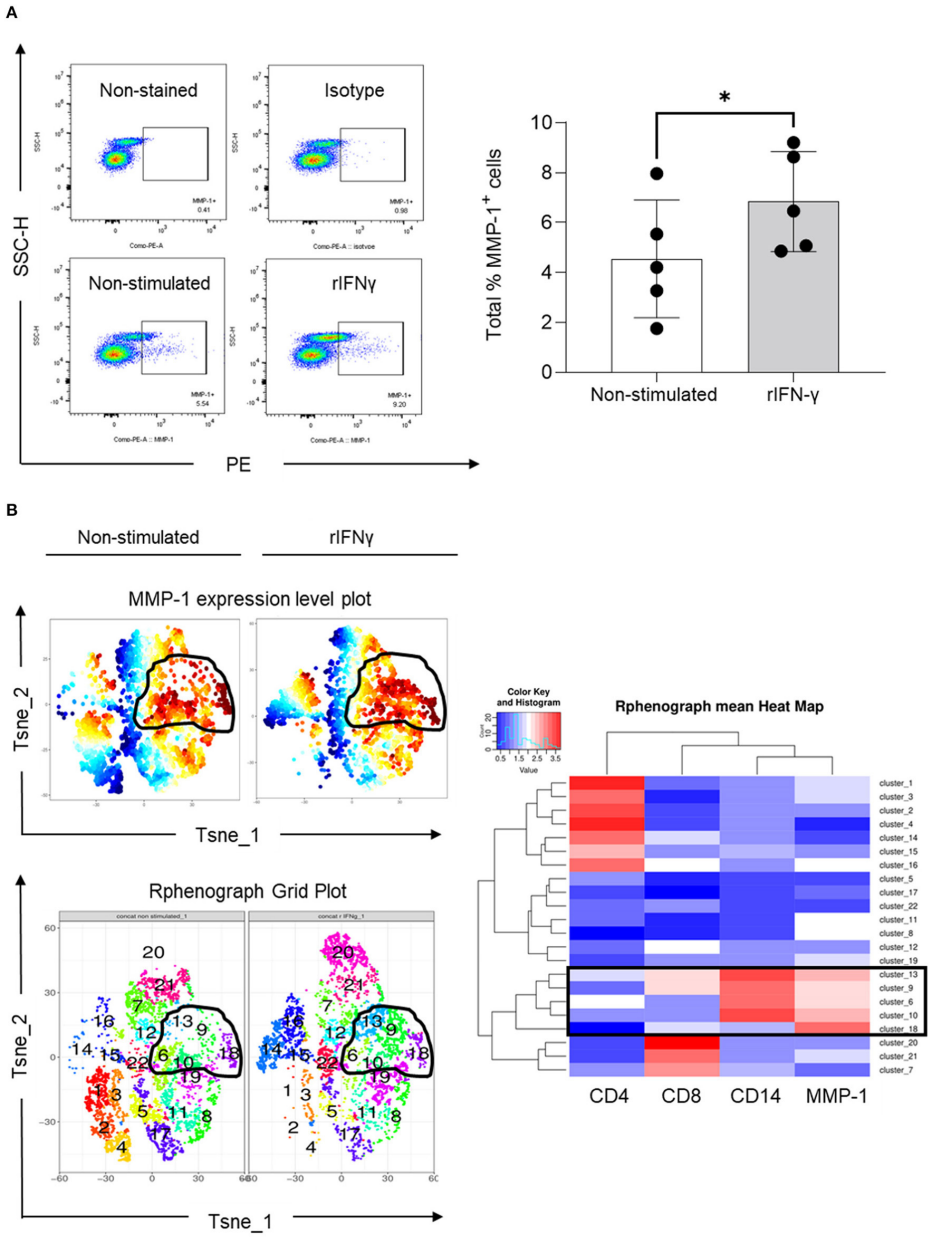
Expression of MMP-1 in peripheral blood mononuclear cells. **(A)** Representative dot plots illustrating the selection of MMP-1 positive gate on non-stimulated and rIFN-γ stimulated cells (bottom panel) using as references non stained and PE-isotype-stained cells (upper panel). Graph shows the individual frequency of total MMP-1 expression in PBMCs (*n* = 5). **(B)** Unsupervised high dimensional analysis of flow cytometry data tSNE showing MMP-1 positive clusters (expression level plot, upper left panel) and matching areas on phonograph grid plot (bottom left). Heat map shows the hierarchical clustering of all identified clusters, according CD4, CD8, CD14 and MMP-1 expression (right panel). *means statistically significant.

To understand whether IFN-γ indeed regulates MMP-1 expression in human blood mononuclear cells, we performed an *in vitro* assay in which PBMCs were stimulated with recombinant IFN-γ (rIFN-γ, 5 ng/ml). Our results indicated that rIFN-γ increased the total number of PBMCs expressing MMP-1 (Figure 5A). Expansion of cells expressing MMP-1 after rIFN-γ stimulation can also be visualized on the MMP-1 expression level plots (Figure 5B, upper panel). We then examined which type of mononuclear leukocytes express MMP-1. Phenograph grid plots, which are generated based on the different expression levels of the different markers used (CD4 and CD8 for T cells, and CD14 for monocytes/macrophages) showed that 6, 9, 10, 13 and 18 were clusters with higher expression of MMP-1 (contoured). Among them, clusters 9 and 13 showed a clearer modulation of MMP-1 expression in non-stimulated vs. stimulated cells (Figure 5B, left panel). As visualized in the heatmap (boxed), MMP-1 positive cells were mainly CD14^+^ cells (clusters 6, 9, 10 and 13). While cluster 18 showed high MMP-1 expression, it was negative for CD4, CD8 and CD14 (Figure 5B, right panel). These cells can be B cells, natural killer cells, or double-negative T cells (CD3^+^CD4^−^CD8^−^), which requires further investigation.

## Discussion

In this study, we aim to identify potential mechanisms favoring leaflet tearing during PMV, a serious, life-threatening complication that usually requires immediate surgical intervention. Of note, study design assessing the structural, cellular, and molecular characteristics of the tissues allowed us to examine overall tissue changes, i.e., not necessarily only the leaflet tear regions. Thus, our findings are not affected by tissue composition in the rupture area only but instead represent the MV as a whole.

**Figure 6:**
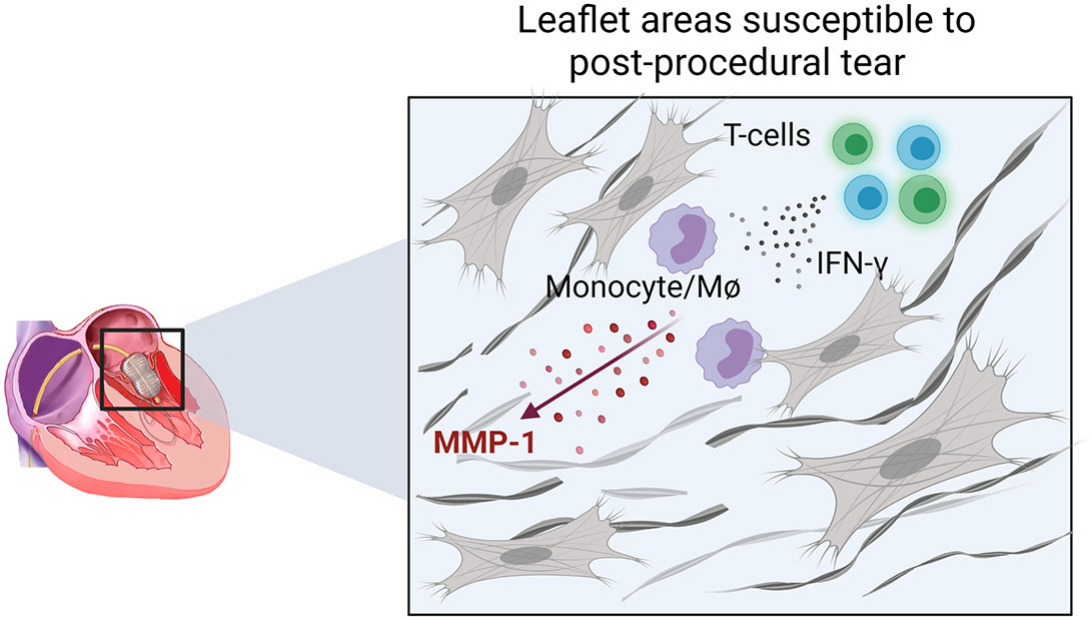
Schematic representation of the cellular and molecular events associated with areas prone to leaflet post-PMV tear. IFN-γ production by T-cells in active inflamed leaflet areas increases the expression of MMP-1 by monocytes/macrophages (MØ) associated with increased collagen degradation, which may lead to a higher susceptibility to laceration.

Previous echocardiographic studies have not been able to identify predictive factors associated with this outcome (17). Our data suggest that leaflet areas with active inflammation are more susceptible to PMV post-procedural leaflet tearing (Figure 6). In these areas, T cells may participate as important adaptive sources of proinflammatory cytokines, including IFN-γ, which stimulates MMP-1 production in monocytes/macrophages. Over time, these inflammatory cells contribute to heterogenous collagen degradation/remodeling resulting in localized leaflet remodeling, characterized by irregular thick and thin collagen layers with the latter likely more susceptible to leaflet laceration (Figure 6).

Patients with severe MS show substantial collagen and elastin leaflet changes, and thus an altered MV leaflet architecture (5, 18). Although we could not find differences in MV leaflet collagen orientation and composition in patients who had leaflet tearing vs. no tearing, we found a distinct layered composition of tissue lacking collagen alternating with fibrous tissue in MVs excised from RHD patients from both groups. These low-density collagen areas are comprised mainly by proteoglycans and glycosaminoglycans (18, 19), abundant components of valvular ECM (20). In addition to the mechanobiological role, the ECM is a source of ligands for cell surface receptors, which transfer mechanical strains to the cells and initiate intracellular signaling pathways (21–23). Notably, we observed aggregates of inflammatory mononuclear cells in the ECM low-density areas, while fibrotic areas with high collagen density showed low cellularity and were generally lacking mononuclear cells.

Inflammation and its impact on mitral leaflet tissue integrity has been shown in several fibrocalcific valvopathy studies (5, 24–26). The evidence supports that inflammation is as mediator of pathological valve remodeling (25, 27). Most studies however have explored aortic valve pathologies, while mitral valve pathobiology remains much less understood (28). To investigate whether inflammation plays a role in rheumatic MS and risk for PMV leaflet tearing, we evaluated the frequency of mononuclear cell types, such as macrophages (CD68^+^) and T cells (T-helper (Th), CD4^+^ and T-cytotoxic, CD8^+^). Although we did not find differences between the “no leaflet tear” and “leaflet tear” groups in terms of inflammatory cell frequencies, we found a moderate positive correlation between the numbers of T-cells (CD4^+^ and CD8^+^) and MV leaflet thickness, indicating a possible contribution of these cells to valve tissue remodeling in the “leaflet tear” group. In a different disease context, T-cells have been associated with less stable and significantly collagen reduced atherosclerotic lesions (29) and exhibit pathogenic roles in several other cardiovascular diseases (30–32).

Proinflammatory T-cells are the main source of IFN-γ and are considered to play an antifibrotic role by suppressing fibroblast-induced collagen synthesis (12, 33–35). Part of this effect seems to be associated with induction of MMPs expression, such as MMP-1, MMP-2, MMP-7, MMP-9, and MMP-13 (12, 36, 37). In the present study, we showed that the “leaflet tear” group has higher expression of IFN-γ and MMP-1 in relation to “no leaflet tear” group. Moreover, we observed a positive correlation between the expression of MMP-1 and IFN-γ in the “leaflet tear,” but not in the “no leaflet tear” group. Immunostaining showed that MMP-1 is focally expressed and located predominantly in areas of high cellularity, which are areas rich in inflammatory cells aggregates. It is reasonable to suggest that these areas are likely to be more susceptible to leaflet tear. MMP-1 is the major human interstitial collagenase and is considered a multifunctional molecule as it participates in the degradation of collagen fibers, and in the cleavage of non-matrix substrates and cell surface molecules, which implies a role for MMP-1 in the regulation of cellular behavior (38–41). Besides the immune cells, it is known that valvular interstitial cells are important source of MMPs and could mediate ECM degradation (7). Activated valvular interstitial cells express excessive levels of MMPs promoting leaflet degeneration in myxomatous heart valves (7). In human atherosclerosis, MMPs expressed by resident smooth muscle cells cause weakening of plaque and favor its rupture (42). Therefore, activated valvular myofibroblast-like cells may also contribute to proteolytic imbalance in patients with post-procedural leaflet tear. Moreover, it has been shown that T-cells can regulate MMP expression in smooth muscle cells (43). Together, these pieces of evidence suggest a potential crosstalk between valvular interstitial cells and leukocytes that may boost the ECM remodeling process.

During carcinogenesis, MMP-1 can mediate metastasis by initiating and sustaining the growth of tumors through the loss of cell adhesion, decreasing apoptosis and deregulating cell division (44). Secreted MMP-1 has been used as a prognostic marker for several types of cancers (45–47). Evaluation of MMP-1 plasma levels in patients with rheumatic MS could also be explored as a prognostic biomarker for leaflet laceration during PMV. Our study shows that MMP-1 expression by CD14^+^ cells (monocytes) are modulated by a proinflammatory environment (IFN-γ). Evaluating MMP-1 expression in circulating CD14^+^ T-cells as well as valvular macrophages (CD68^+^) in patients with rheumatic MS could help to better understand the impact of these cells on PMV complications. Expression of MMPs in lymphocytes has been previously reported for MMP-1, MMP-2 and MMP-9 (48, 49). Moreover, MMP expression in monocytes and macrophages was shown to be related to atherosclerotic plaque instability and myocardial infarction (50). Increased levels of MMPs during monocyte/macrophage activation seem to be depended on mitogen activated protein kinases, phosphoinositide-3-kinase and inhibitor of κB kinase-2 (51). Our research group has shown that inflammation and its downstream events are the key factors in mitral heart valve disease and could serve as targets for regenerative medicine (25, 26, 52, 53).

## Summary

Our study reports on histopathological, cellular, and molecular changes that potentially contribute to MV tearing during PMV. We provide insights into a synergistic mechanism between MMP-1 and IFN-γ generating an active inflammatory leaflet tissue response contributing to the localized degradation of collagen predisposing to leaflet tear (Figure 6). Future work should explore if plasma MMP-1 levels can be a prognostic biomarker predicting increased leaflet laceration risk, and if medical therapy can stabilize leaflet tissue inflammation prior to PMV.

## Data Availability Statement

The raw data supporting the conclusions of this article will be made available by the authors, without undue reservation.

## Ethics Statement

The studies involving human participants were reviewed and approved by Certificado de Apresentação de Apreciação Ética (CAAE) protocol #: 32715214.9.0000.5149. The patients/participants provided their written informed consent to participate in this study.

## Author Contributions

LSAP, DB-G, WD, MP, and EA contributed to conception and design of the study. LSAP, T-DL, and DB-G performed experiments and statistical analysis. CG, LA, CG, DR, and WE contributed to bio specimens and clinical data collection. LSAP wrote the manuscript. DB-G wrote sections of the manuscript. JD-B, RL, LGP, JH, MA, MN, WD, and EA edited and critically revised manuscript. RL and EA provided critical input during the project and contributed to overall project supervision and funding. All authors approved the submission of the manuscript.

## Funding

This work was supported by National Institutes of Health research Grants R01 HL147095 and R01 HL136431 to EA and R01 HL141917 to RL and EA.

## Conflict of Interest

The authors declare that the research was conducted in the absence of any commercial or financial relationships that could be construed as a potential conflict of interest.

## Publisher's Note

All claims expressed in this article are solely those of the authors and do not necessarily represent those of their affiliated organizations, or those of the publisher, the editors and the reviewers. Any product that may be evaluated in this article, or claim that may be made by its manufacturer, is not guaranteed or endorsed by the publisher.

## Supplementary Material

Supplementary Figure 1 Quantitative analysis for immunohistochemistry staining for CD68, CD8, and CD4 stenotic MVs. Graph shows the frequency of each cell subset from patients in “no leaflet tear” group (blue, *n* = 8) and in “leaflet tear” group (red, *n* = 8). Scale bar = 200 mm.
