# Supplementary figures and images for: Proinflammatory Matrix Metalloproteinase-1 Associates With Mitral Valve Leaflet Disruption Following Percutaneous Mitral Valvuloplasty
Source: Front Cardiovasc Med. 2022 Jan 20;8:804111. doi: 10.3389/fcvm.2021.804111 (PMC8811173; doi:10.3389/fcvm.2021.804111)

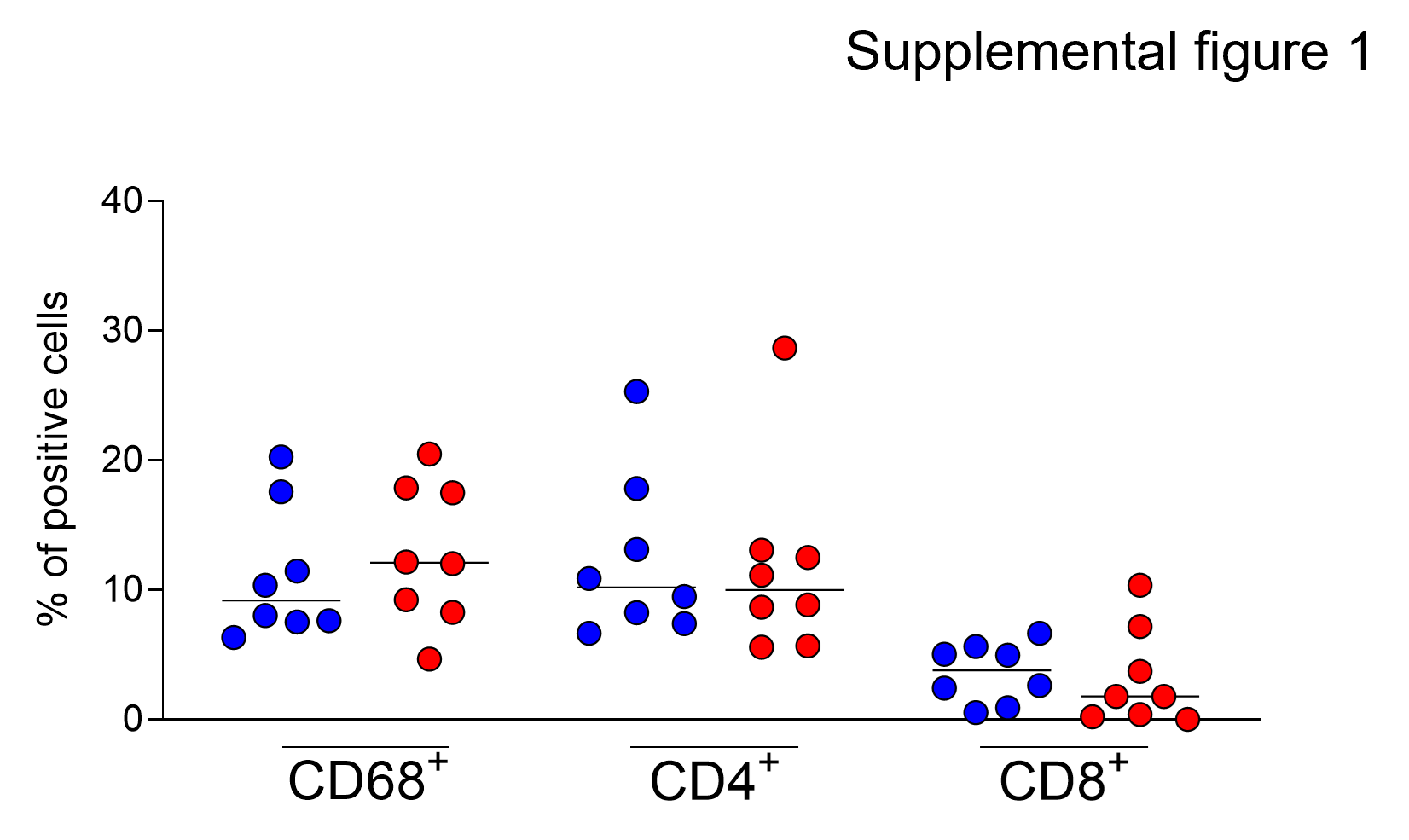

Supplement: Supplementary Figure 1 — Quantitative analysis for immunohistochemistry staining for CD68, CD8, and CD4 stenotic MVs. Graph shows the frequency of each cell subset from patients in “no leaflet tear” group (blue, n = 8) and in “leaflet tear” group (red, n = 8). Scale bar = 200 mm. [file Image_1.TIF]
